# Miscarriages in families with an offspring that have classic congenital adrenal hyperplasia and 21-hydroxylase deficiency

**DOI:** 10.1186/s12884-018-2091-8

**Published:** 2018-11-23

**Authors:** Helmuth G. Dörr, Johannes Hess, Theresa Penger, Michaela Marx, Patricia Oppelt

**Affiliations:** 1 Division Pediatric Endocrinology, Department Pediatrics, University Hospital of Erlangen (Friedrich Alexander-Universität Erlangen-Nürnberg), Loschgestr. 15, 91054 Erlangen, Germany; 2 Pediatric Gynecology, Department. Gynecology and Obstetrics, University Hospital of Erlangen, Erlangen, Germany

**Keywords:** Classic CAH, Miscarriage rate, Offspring with CAH

## Abstract

**Background:**

The most common form of congenital adrenal hyperplasia is 21-hydroxylase deficiency (CAH). Both men and women with classic CAH have lower fertility rates than the general population, and an increased rate of miscarriages has been reported in affected women. There are no data on the incidence rate of miscarriages in families with an offspring that have classic CAH.

**Methods:**

We studied families with a history of classic CAH. The families came from different parts of Germany and attended the annual meeting of the German CAH support group for parents and patients which was held in Hamburg in September 2014. The data was collected anonymously by a paper-based questionnaire which was completed by the families at home. The families also accepted the responsibility to address this question to their siblings. In all, the data of 50 families with at least one child with classic CAH, and the data of 164 parental siblings were available for evaluation. Miscarriage rates were calculated in relation to the reported pregnancies.

**Results:**

Twenty-two miscarriages were reported from 19 families. At least one miscarriage occurred in 38% of the families, three families experienced two miscarriages and 16 families had one miscarriage each. The mean miscarriage rate was 15.8%. The heterozygous mothers had a total of 90 siblings (41 m, 49 f), while 74 siblings (33 m, 41 f) were reported from the heterozygous fathers. The miscarriage rate was 10.1% in the families of the mothers` siblings, and 11.4% in the families of the fathers` siblings. The genotype was known in all parents that have an offspring with classic CAH, but not defined in 82% of the maternal siblings, and in 86% of the paternal siblings. No child with classic CAH has been diagnosed in any of the sibling’s families to date.

**Conclusion:**

Our data show that the miscarriage rate in German families with a child with classic CAH is not elevated.

**Electronic supplementary material:**

The online version of this article (10.1186/s12884-018-2091-8) contains supplementary material, which is available to authorized users.

## Background

Miscarriage is the most common complication of early pregnancy. Among women with known pregnancy, the miscarriage rate is about 10 to 20% [1–4]. For a prospective cohort of couples from Denmark, recruited prior to conception, the mean incidence was 17% [5]. A study from the USA calculated in a cohort of 344 couples a pregnancy loss of 28% which was connected to various preconception lifestyle factors [6]. Overall, there are a variety of factors associated with an increased risk for miscarriage including the age of the mother and father, lifestyle factors, and medical conditions such as infections, fetal and maternal diseases [7–10]. Recently, subclinical hypothyroidism was identified as an endocrine risk factor in women before 20 weeks of pregnancy [11].

The most common form of congenital adrenal hyperplasia is 21-hydroxylase deficiency (CAH) due to mutations in the active gene CYP21A2 [12]. The classic form of CAH with salt wasting (SW) or simple virilizing (SV) presents at childbirth, whereas the nonclassic form of CAH (NCAH) is generally diagnosed after adolescence. In women with classic CAH, the fertility rate is lower than in the general female population [13, 14], and an increased rate of miscarriages of 36% has been reported in Germany [15] The miscarriage rate in women with NCAH is somewhat lower than in women with classic CAH. The reported rates vary between 19% in France [16, 17] and 20.3% in Israel, where this rate was significantly higher than the 10.9% rate in the general population [18].

To the best of our knowledge, we found no data on the incidence rate of miscarriages in families with an offspring with classic CAH when both parents are heterozygous gene carriers. Therefore, the objective of this paper was to examine the risk of miscarriages in those families.

## Methods

We studied families with an offspring that have classic congenital adrenal hyperplasia due to 21-hydroxylase deficiency (CAH). The families came from different parts of Germany and attended the annual meeting of the German CAH support group for parents and patients which was held in Hamburg in September 2014. Verbal consent was obtained from all families. The participation at the study was voluntarily. The mothers and the fathers of the participating families also accepted the responsibility to address this question to their siblings. All parental siblings were included in the study, i.e. also single siblings, married siblings and siblings with children without being married. The data was collected anonymously by a paper-based questionnaire (Additional file 1) in accordance with the Declaration of Helsinki. We had no data on the ages of the children and the individual *CYP21A2* mutations. The study was approved by the local ethics committee of the Dept. Pediatrics of Erlangen. The questionnaire was completed by the families at home and sent to Erlangen (HGD), where the data was analyzed.

In all, the data of 50 families with at least one child with classic CAH, and the data of 164 parental siblings were available for evaluation. A spontaneous abortion after known pregnancy by the mother was defined as miscarriage. Miscarriage rates were calculated and compared to expected values in the general population. Statistical analysis was performed using SPSS. One-way ANOVA was used to compare miscarriage rates among the parents, sibs of heterozygous females, and sibs of heterozygous males.

## Results

The 50 families had a total of 117 children, including 67 children (57.3%) with classic CAH (39 girls, 28 boys). There were no children with nonclassic CAH. Most families (73%) only had a single child with classic CAH. Twelve families had two affected children, and one family had six children with classic CAH. The age of the parents ranged from 22 to 55 years for the mothers and from 25 to 60 years for the fathers. The male siblings had ages between 18 and 63 years, and the female siblings between 28 and 65 years.

Twenty-two miscarriages, distributed over 19 families, were reported from the families, while 31 families had no miscarriage. This means that at least one miscarriage occurred in 38% of the interviewed families, three families experienced two miscarriages and 16 families had one miscarriage each. Considering the number of reported miscarriages in relation to the number of clinically recognized pregnancies in the families (*n* = 139), the miscarriage rate was 15.8%.

The genotype was known in all parents that have an offspring with classic CAH, but the parents could not describe the individual mutations. We assume that the children might have genotypes associated with groups null or A, since all children had the severe form of CAH with salt wasting. The genotype was not defined in 82% of the maternal siblings, and in 86% of the paternal siblings.

The heterozygous mothers had a total of 90 siblings (41 brothers, 49 sisters), while 74 siblings (33 brothers, 41 sisters) were reported from the heterozygous fathers. In the siblings with children, the reported miscarriage rate was 10.1% in the families of the mothers` siblings, and 11.4% in the families of the fathers` siblings. No child has been diagnosed with classic CAH in any of these families to date.

## Discussion

Women with classic CAH have lower fertility rates compared to general population [13, 14, 19, 20]. Recently, this finding was also reported in men with classic CAH [21]. In women, the causes were assigned to factors such as adrenal overproduction of androgens and progestins, neuroendocrine factors, genital surgery and/or psychosocial factors [22]. However, at this point, one should not confuse the terms fertility and fecundity. Fertility is the natural capability to produce offspring, whereas fecundity is the potential for reproduction of a population. Traditionally, low pregnancy rates have been reported especially in women with classic CAH and the salt wasting form [23, 24], but recent studies show that the pregnancy rate for all women with classic CAH who actively tried to conceive was not different from that in the normal population, provided a good biochemical control with optimized glucocorticoid and mineralocorticoid substitution regimens [19, 25].

Data on an increased rate of miscarriages in women with classic CAH is inconsistent in the literature and varied from 10% [13, 23] to 36% [15]. Women with nonclassic congenital adrenal hyperplasia (NCAH) have also a high rate of miscarriages. The rate of 20.3% in Israel was higher than in the general population [18], but similar to data reported in France before the diagnosis of NCAH [17]. It is still not clear why a higher miscarriage rate was found in women with classic CAH and also in women with NCAH. There are many assumptions circulating in the literature but no clear facts. The severity of classic CAH might play a role because Krone et al. identified in their cohort one woman with classic CAH and salt wasting who had two spontaneous abortions before successful pregnancy [20]. Factors contributing to the lower fertility rate might play a role, too. In particular, adrenal overproduction of androgens due to inadequate glucocorticoid and mineralocorticoid substitution may cause a high rate of spontaneous abortions [22]. In France, the rate of miscarriages in NCAH pregnancies decreased to 10% in women in whom treatment was started before pregnancy [17] suggesting that therapy may reduce the rate of pregnancy loss in NCAH [26].

Patients with classic CAH suffer from a considerable lifelong burden due to the need for regular physician visits, the risk of adrenal crisis and the long-term impacts of CAH-associated symptoms and treatment [27]. This conclusion applies also for families with a child with classic CAH. Moreover, if a family with a child with CAH decided on a new pregnancy, then the obligate heterozygous pregnant woman might experience more stress than a normal healthy pregnant woman and must be managed by high-risk practitioners [25, 28]. Reports in the literature show that prenatal stress could be associated with higher rates of spontaneous abortion [29–31]. However, a large study from the USA found no clear pattern of association between stress salivary cortisol and incident pregnancy loss [32].

Overall, data on miscarriages rate in families with a child with classic CAH when both parents are obligate heterogeneous gene carriers are missing in the literature. We could show that the average miscarriage rate of 15.8% in families with a classic CAH child was not different from the rate found in a general population. The miscarriage rates in the siblings´ families were lower, but the differences were statistically not significant. There were some limitations to our study. The findings are limited by the nature of the data. The population studied is a selective group of affected families. The data were obtained by a questionnaire from CAH families who attended the annual meeting of the German CAH Support Group for Patients and Parents, thus the figure of miscarriages might not be representative.

## Conclusions

Our data show that the rate for miscarriages compared to published data in a general population is not elevated, neither in the families with a classic CAH child nor in the families of the siblings.

## Additional file

Additional file 1: Translated Questionnaire from German Families with an affected child with classic CAH. (DOCX 15 kb)

## Abbreviations

CAH: Congenital adrenal hyperplasia
CYP21A2: Cytochrome P450 family 21 subfamily A member 2
F: Female
M: Male
NCAH: Nonclassic congenital adrenal hyperplasia
