# Supplementary material for: Miscarriages in families with an offspring that have classic congenital adrenal hyperplasia and 21-hydroxylase deficiency
Source: BMC Pregnancy Childbirth. 2018 Nov 23;18:456. doi: 10.1186/s12884-018-2091-8 (PMC6251199; doi:10.1186/s12884-018-2091-8)
Supplement: Supplementary file 1 — Translated Questionnaire from German Families with an affected child with classic CAH. (DOCX 15 kb) [file 12884_2018_2091_MOESM1_ESM.docx]

**Additional file 1: Translated Questionnaire from German**

**Families with an affected child with classic CAH**

Zip Code:

Age Mother: Age Father:

Miscarriages: o yes o no If yes, number of miscarriages:

Number of known pregnancies:

Number of healthy children:

Number and sex of offspring with classic CAH:

**Familiy members of the mother:**

| Sex  (m, f) | Age  (yrs) | Known Genotype  (yes, no) | Number  of known pregnancies | Number of miscarriages |
| --- | --- | --- | --- | --- |
|  |  |  |  |  |
|  |  |  |  |  |
|  |  |  |  |  |

**Familiy members of the father:**

| Sex  (m, f) | Age  (yrs) | Known Genotype  (yes, no) | Number  of known pregnancies | Number of miscarriages |
| --- | --- | --- | --- | --- |
|  |  |  |  |  |
|  |  |  |  |  |
|  |  |  |  |  |

Please send the completed questionnaire to

Prof. Dr. HG Dörr, Dept. Pediatrics, University Hospital Erlangen

Loschgestr. 15, 91054 Erlangen either by mail or by fax 09131-853-6131.
